# MicroRNA-34a targets epithelial to mesenchymal transition-inducing transcription factors (EMT-TFs) and inhibits breast cancer cell migration and invasion

**DOI:** 10.18632/oncotarget.15214

**Published:** 2017-02-09

**Authors:** Saber Imani, Chunli Wei, Jingliang Cheng, Md. Asaduzzaman Khan, Shangyi Fu, Luquan Yang, Mousumi Tania, Xianqin Zhang, Xiuli Xiao, Xianning Zhang, Junjiang Fu

**Affiliations:** ^1^ Key Laboratory of Epigenetics and Oncology, Research Center for Precision Medicine, Southwest Medical University, Luzhou, Sichuan, China; ^2^ Chemical Injuries Research Center, Baqiyatallah Medical Sciences University (BMSU), Tehran, Iran; ^3^ State Key Laboratory of Quality Research in Chinese Medicine, Macau University of Science and Technology, Macau (SAR), China; ^4^ Faculty of Applied Sciences, Ton Duc Thang University, Ho Chi Minh City, Vietnam; ^5^ The Honors College, University of Houston, Houston, TX, USA; ^6^ Division of Computer Aided Drug Design, Red-Green Computing Centre, Dhaka, Bangladesh; ^7^ Pathology Department, Southwest Medical University, Luzhou, Sichuan, China; ^8^ Department of Cell Biology and Medical Genetics, Zhejiang University School of Medicine, Hangzhou, Zhejiang, China

**Keywords:** microRNA-34a, thymoquinone, epithelial-mesenchymal transition, metastasis, breast cancer

## Abstract

MicroRNA-34a (miR-34a) plays an essential role against tumorigenesis and progression of cancer metastasis. Here, we analyzed the expression, targets and functional effects of miR-34a on epithelial to mesenchymal transition-inducing transcription factors (EMT-TFs), such as TWIST1, SLUG and ZEB1/2, and an EMT-inducing protein NOTCH1 in breast cancer (BC) cell migration and invasion and its correlation with tumorigenesis and clinical outcomes. Expression of miR-34a is downregulated in human metastatic breast cancers (MBC) compared to normal breast tissues and is negatively correlated with clinicopathological features of MBC patients. Ectopic expression of miR-34a in MBC cell-line BT-549 significantly inhibits cell migration and invasion, but exhibits no clear effect on BC cell growth. We found that miR-34a is able to inactivate EMT signaling pathway with mediatory of NOTCH1, TWIST1, and ZEB1 upon 3′-UTR activity in MBC cell lines, but has no inhibitory effects on SLUG and ZEB2. Furthermore, we investigated the synergistic effects of Thymoquinone (TQ) and miR-34a together on the expression of EMT-associated proteins. Results showed that co-delivery of miR-34a and TQ is able to inactivate EMT signaling pathway by directly targeting TWIST1 and ZEB1 in BT-549 cell line, indicating that they might be a promising therapeutic combination against breast cancer metastasis. Epigenetic inactivation of the EMT-TFs/miR-34a pathway can potentially alter the equilibrium of these regulations, facilitating EMT and metastasis in BC. Altogether, our findings suggest that miR-34a alone could serve as a potential therapeutic agent for MBC, and together with TQ, their therapeutic potential is synergistically enhanced.

## INTRODUCTION

From historical to contemporary science, metastatic breast cancers (MBC) have become one of the deadliest female malignancies worldwide with >41,000 deaths per year [1, 2]. Despite significant progresses in clinical treatment strategies of MBC, the 5-year survival rate after curative resection is reported to be only ∼24% [3]. Chemotherapy, radiotherapy and molecularly targeted therapy are the main components of MBC therapy [4]. The clinically heterogeneous nature of MBC relatively arises from its biological and genetic heterogeneity. Therefore, a better understanding of the genetic and molecular characteristics of MBC is warranted to decrease mortality and improve patient's quality of life [5]. A variety of genetic and epigenetic abnormalities characterizes the development of the metastatic process, where epithelial to mesenchymal transition (EMT) plays a vital, supportive role for local invasion, migration, growth, and drug resistance of breast cancer (BC) cells [6, 7].

EMT is characterized by the loss of epithelial cell polarity and tight cell-cell junction, such as E-cadherin and β-catenin, leading to the enhancement of the migratory and invasive attributes and the appearance of mesenchymal biomarkers, such as N-cadherin and vimentin [8, 9]. EMT is modulated by different extracellular signals and several activating transcription factors, such as members of the SNAIL, zinc-finger E-box-binding (ZEB) and basic helix-loo-helix (bHLH) families, including zinc finger protein SNAI2 (SLUG), Twist-related protein 1 (TWIST1), ZEB1 and ZEB2, which are master coordinators of EMT and the metastatic program [10–13]. The EMT-inducing transcription factors (EMT-TFs) induce EMT responses during tumor metastases by binding to the E-box sequences in the proximal promoter region of the repress epithelial genes and recruiting another post-translational modifications bio-machines [9, 10, 14]. In the early stages of MBC, EMT-TFs, like TWIST1, ZEB1/2, and SLUG, repress E-cadherin and β-catenin expression; promote EMT, cell motility and invasiveness; and permit the intravasation of tumor cells [15–18].

Recently, microRNA-34a (miR-34a) was identified as a novel class of tumor-suppressive miRNAs that is downregulated in several human cancers, including breast, lung, and liver; re-introduction of miR-34a mimics the inhibition of cancer cell growth and metastasis [19–21]. miR-34a resides on the chromosomal locus 1p36.23 and is involved directly and indirectly with many different oncogenic processes, including growth, survival, differentiation, proliferations, migration, invasion, and immune responses [20]. Accordingly, overexpression of miR-34a acts as a mediator of tumor suppression by transcriptionally regulating TP53, NOTCH, and transforming growth factor beta (TGF-β) signaling pathways [14, 20, 22–25]. So far, it is unclear whether miR-34a epigenetically silences EMT by binding to EMT-TFs in BC. Previous studies have shown that downregulation of TWIST1, SNAIL1, and SLUG has a key role in aggregating MBC treatment and increasing EMT-TFs levels correlated with poor prognosis in a MBC [11, 14, 26].

Evidence implies that miR-34a is a promising non-invasive biomolecular tool for gene regulation in the metastatic process by coordinating TP53, NOTCH1, and TGF-β-pathways [27–29]. Since different cancer cell types display differences in the expression and relevance of EMT regulators [30–32], current attention is focused on systematically analyzing EMT-TFs’ regulatory mechanism and EMT-TFs/miR-34a feedback loop, like the role of TWIST1, SLUG, ZEB1/2 and SNAIL1 in promoting BC migration, invasion and metastasis.

We aimed to investigate the role of miR-34a in human breast tumors with a focus on MBC. Here, we found that miR-34a potentially inhibits TFs, like TWIST1 and ZEB1, and EMT-associated protein NOTCH1 expression upon the 3′-untranslated region (3′-UTR) in BC cells. Transient transfection of miR-34a significantly inhibits cell proliferation, cell migrations, cell survival and cell invasion of BC cell lines by targeting NOTCH1, ZEB1, and TWIST1. We have consequently revealed that miR-34a is downregulated in MBC tissues and suppresses cancer metastasis by affecting several malignancy endpoints.

## RESULTS

### MicroRNA-34a expression in human BC cell lines and tissues

**Figure 1:**
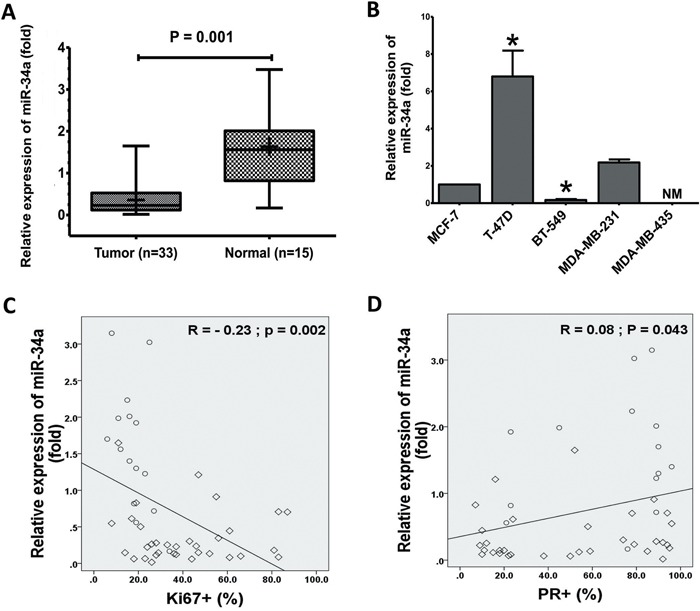
Expression of miR-34a in BC cell lines and BC specimens **A**. The expression levels of miR-34a in normal breast significantly decreased in human cancer tissues that measured by TaqMan® MicroRNA assays kit. **B**. Total RNA was prepared from the cell lysates and expression of miR-34a was quantified by TaqMan MicroRNA Assays. The expressions of miR-34a was normalized against the expression level of U6 snRNA. The expression levels T-47D, BT-549, MDA-MB-231, and MDA-MB-435 were expressed as relative to the miR-34a expression level of MCF-7 cells, non-metastatic BC cell lines (*mean±SD*). Inverse relationship between levels of miR-34a in 48 human breast specimens with Ki67 **C**. PR expression **D**. The diamond indicates human BC specimens (n = 33), and circles represent paired adjacent normal tissue (n = 15). Data were analyzed by Spearman's rank correlation coefficient. In general, it was observed significant correlation between the miR-34a expression levels and some clinicopathological features of MBC patients. Data presented is a representative of three different experiments. (NM, No measured value; * *P<0.05*).

Previous investigational screenings for miR-34a expressions in different tissues and cancer cell types found that the expression of miR-34a is variable in BC [30–32]. Therefore, we first measured the level of mature miR-34a in our collected MBC tissues and human BC cell lines using quantitative reverse transcriptase-PCR (qRT-PCR). Interestingly, the average level of miR-34a expression was consistently and significantly lower in tissues of MBC (*n* = 33) than that of normal breast samples (*n* = 15) (Figure 1A). In addition, miR-34a levels were significantly lower in some MBC cell lines in comparison to non-metastatic BC cell line MCF-7 (Figure 1B). There was no expression of the miR-34a in the MDA-MB-435 BC cells. The above data showed that miR-34a is significantly downregulated in MBC tissues, suggesting that the downregulation of miR-34a most likely affects the progression of MBC.

### MicroRNA-34a expression correlates to clinicopathological features

The demographic and clinical characteristics of the 48 subjects were showed in Table 1. The mean ages of these patients were 46.34 ± 9.71 (age range, 26-67 years). There was no statistically significant difference between the groups in terms of age and number. The relative expression of miR-34a in MBC tissues was significantly related to certain clinicopathological features, like progesterone receptor and Ki-67 proliferation marker status. Furthermore, miR-34a expression levels were inversely correlated with Ki-67 (*r* = −0.23, *P* = 0.002; Figure 1C). Figure 1D presents a positive correlation between the miR-34a expression level ratios and PR. However, clinicopathological features, such as human epidermal growth factor receptor-2, estrogens receptor, and TP53, did not exhibit a significant association with miR-34a expression (Supplementary Figures 1A-1C). In general, a significant correlation between miR-34a expression and clinicopathological features of MBC patients was observed. Although there is no statistical significance observed currently, further research might exhibit a significant correlation between miR-34a expression level and tumor size (p value is 0.08) as well as histological grade (p value is 0.075) if we increase the sample size.

**Table 1:** Relationship between miR-34a expression level and clinicopathological features of MBC patients

Demographic and Histopathologic variables (n)	miR-34a expression levels		P value
	miR-34a^+^ (≥0.74)	miR-34a^−^ (<0.74)	
Subjects (%)	17 (35.4)	31 (64.6)	0.001
Age (yrs.)			
< mean (27)	10 (20.8)	17 (35.4)	0.517
≥ mean (21)	7 (14.6)	14 (29.2)	
Tumor histology			
Ductal (30)	3 (9.1)	27 (80.1)	0.238
Lobular (3)	1 (3.0)	2 (6.1)	
Tumor Size			
≤ 3 cm (4)	2 (6.1)	2 (6.1)	0.08
3-9 cm (24)	2 (6.1)	22 (66.6)	
≥ 9 cm (5)	0 (0)	5 (15.2)	
Diagnosis stage (TNM)			
Stage II (15)	0 (0)	15 (45.5)	0.075
≥ Stage III (18)	4 (12.1)	14 (42.4)	
Node status			
Negative (26)	3 (8.6)	23 (65.7)	0.162
Positive (9)	3 (8.6)	6 (17.1)	
Neoadjuvant therapy (%)			
Yes (12)	3 (9.4)	9 (28.1)	0.399
No (20)	3 (9.4)	17 (53.1)	
Chemotherapy			
Yes (14)	3 (9.4)	11 (34.4)	0.541
No (18)	3 (9.4)	15 (46.9)	
PR			
Negative (22)	6 (12.5)	16 (33.3)	0.02
Positive (26)	11 (22.9)	15 (31.2)	
ER			
Negative (18)	4 (8.3)	14 (29.2)	0.121
Positive (30)	13 (27.1)	17 (35.4)	
P53			
Negative (34)	13 (32.5)	21 (52.5)	0.60
Positive (6)	2 (5.0%)	4 (10.0)	
Ki67			
≤ 30 (27)	14 (29.2)	13 (27.1)	0.03
> 30 (21)	3 (6.2)	18 (37.5)	
Her2/neu			
Low/weak (27)	10 (20.8)	17 (35.4)	0.517
Moderate/strong (21)	7 (14.6)	14 (29.2)	

Abbreviations: miR-34a, MicroRNAs-34a; TNM, TNM classification of malignant tumors; ER, estrogens receptor; PR, progesterone receptor; HER2, human epidermal growth factor receptor-2.

Data are presented as a number for all others. %, percent of total samples.

### MicroRNA-34a inactivates the EMT-TFs signaling in BC cell lines

**Figure 2:**
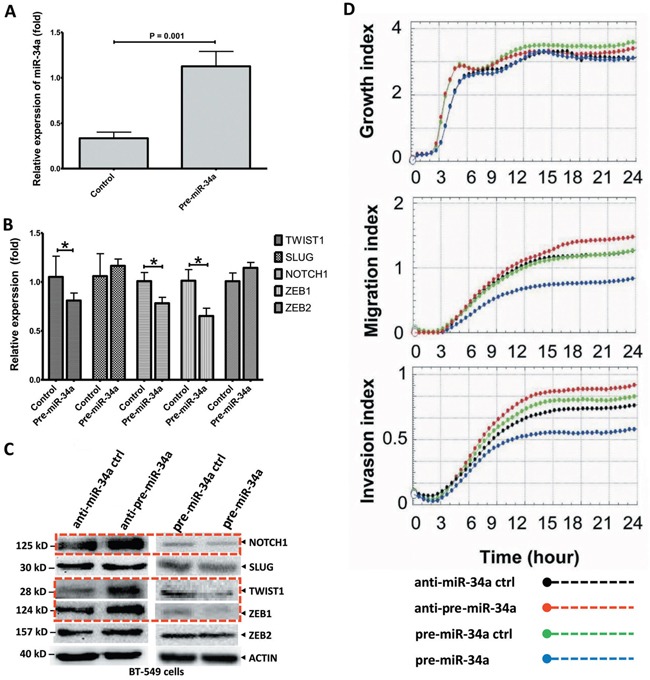
Ectopic expression of miR-34a inhibits the cell migration and invasion in BT-549 BC cells **A**. The BT-549 cells were transfected with plasmid pre-miR-34a and pre-miR-34a-controls as indicated. The miR-34a expression which measured by stem-loop qRT-PCR in transfected cells was significantly increased. **B**. The relative expression of EMT-TFs in BT-549 cells transfected with miR-34a mimic were analyzed by qRT-PCR. **C**. Cells were treated the same as above and protein lysis were further analyzed by Western blot assay for the protein expression of EMT-TFs. The over-expressed miR-34a cells reduced NOTCH1, TWIST1, and ZEB1 levels. Nevertheless, the SLUG and ZEB2 levels are no change. **D**. miR-34a dependently inhibited, migration and invasive characteristics of BT-549 cells. However, no clear effect on cancer cell growth. All data were showed with mean ± SD. Similar results were obtained from three independent experiments. The qRT-PCR results normalized to 18S RNA (18S) or U6 snRNA (U6). In Western blot, the β-actin (ACTIN) as internal controls. (* *P<0.05*).

The qRT-PCR and Western blot analysis were conducted to investigate whether miR-34a expression could remarkably affect the EMT-TFs SNAIL1, SLUG, TWIST1, ZEB1 and ZEB2, and NOTCH1 expression levels. As shown in Figure 2A, the miR-34a expression in BT-549 cells transfected with miR-34a mimic was significantly increased. Upon overexpression, miR-34a reduced NOTCH1, TWIST1, and ZEB1 levels, although it expressed a less significant reduction in their corresponding protein levels (*P* > 0.05; Figures 2B-2C), indicating both transcriptional and post-transcriptional regulations. However, overexpression of miR-34a did not substantially affect SLUG, ZEB2 expression (*P* ≥ 0.05; Figures 2B-2C) and SNAIL1 expression (data not shown). Consistently, after the inhibition of miR-34a, TWIST1, NOTCH1 and ZEB1 protein levels were significantly increased. (Figure 2C). All these results suggested that miR-34a is able to inactivate the EMT signaling pathway with mediation of NOTCH1, TWIST1 and ZEB1 in MBC cell lines.

### MicroRNA-34a inhibits migrations and invasion in BC cells *in vitro*

To investigate miR-34a's effects on BC cell growth, migration, and invasion, pre-miR-34a for the overexpression of precursor miR-34a (pre-miR-34a), or anti-miR-34a for the overexpression or inhibition of miR-34a, were transiently transfected into the MBC cell lines, BT-549 (Figure 2D) and MDA-MB-435 (Supplementary Figure 2), respectively. Our results clearly showed that miR-34a's expression did not significantly inhibit cell proliferation in BT-549 cells. Pre-miR-34a restoration inhibits cell migration and invasion in comparison to the pre-controls. Up-regulated miR-34a contributed to a significant down-regulation of cell migration and invasion. Strikingly, the inhibited-miR-34a in BT-549 BC cells showed more significant migrations and invasions after 12 h of cell culture. Here, we did not observe significant changes in cell migration in MDA-MB-435 cell lines with anti-miR-34a overexpression (Supplementary Figure 2); thus, we concluded that miR-34a functions are dependent on MBC cell line types. Therefore, miR-34a suppresses several malignancy parameters in human breast tumor cells, but has no obvious effects on cancer cell growth.

### Evaluation of EMT status and miR-34a in BC cell lines

**Figure 3:**
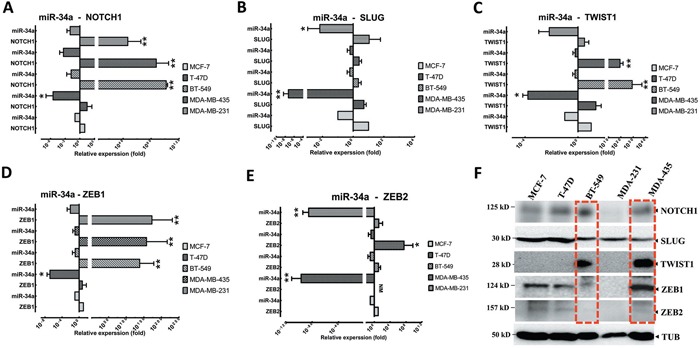
EMT-TFs demonstrate high expression levels in MBC cell lines in front of miR-34a expression Relative expression levels of NOTCH1 **A**., SLUG **B**., TWIST1 **C**., and ZEB1 **D**. and ZEB2 **E**. were detected in non-metastatic BC cell lines (MCF7 and T47D), and MBC cell lines (MDA-MB-231, MDA-MB-435 and BT-549), in comparison to relative expression levels of miR-34a using qRT-PCR. **F**. EMT-TFs expression level was detected in MBC cell lines by western blot. Totally, EMT-TFs were overexpressed in invasive BC cell lines. Similar results were obtained from three independent experiments. Horizontal bars show with mean±SD. The Tubulin (TUB) in western blot assay, and 18S or U6 in qRT-PCR as controls. (* *P<0.05* and ** *P<0.001* versus MCF-7 group).

Although previous studies have demonstrated that EMT-TFs played a fundamental role in the initiation, aggregation, progression, and metastasis of BC by inducing the activation of NOTCH1, TP53, and TGF-β cascade [10, 13, 33], EMT-TFs’ expression patterns, in comparison to miR-34a, have not been well characterized in human BC. To assess whether EMT-TFs and NOTCH1 are important mediators of metastatic potential in BC cells, we first evaluated NOTCH1, TWIST1, SLUG, and ZEB1/2 expression in a panel of established metastatic and non-metastatic BC cell lines (Figure 3). NOTCH1, SLUG, TWIST1, and ZEB1/2 expression levels, in contrast to miR-34a, were verified in BC cell lines, which confirmed that NOTCH1 and EMT-TFs partially inhibit miR-34a expression in cells (Figures 3A-3E). Altogether, these data suggested that NOTCH1 (Figure 3A), TWIST1 (Figure 3C), and ZEB1 (Figure 3D) are reverse correlated with miR-34a in the both MBC and BC cell lines (especially BT-549 and MDA-MB-435 cell lines) (P≤0.001). However, the relative expression of the SLUG and ZEB2 and miR-34a expression was not significantly co-related in BC cell lines (Figures 3B and 3E, respectively). In addition, the EMT-TFs protein expression was higher in both MBC cell lines, MDA-MB-435 and BT549 in comparison to non-metastatic BC cell lines (Figure 3F). In considering both gene and protein levels, TWIST1, SLUG, and ZEB1 expressions were higher compared to the lower expression of miR-34a. These results suggested that the expression of EMT-TFs positively correlates with the metastatic potential of BC cell lines.

### EMT-TFs overexpression in malignant progression of BC tumors

**Figure 4:**
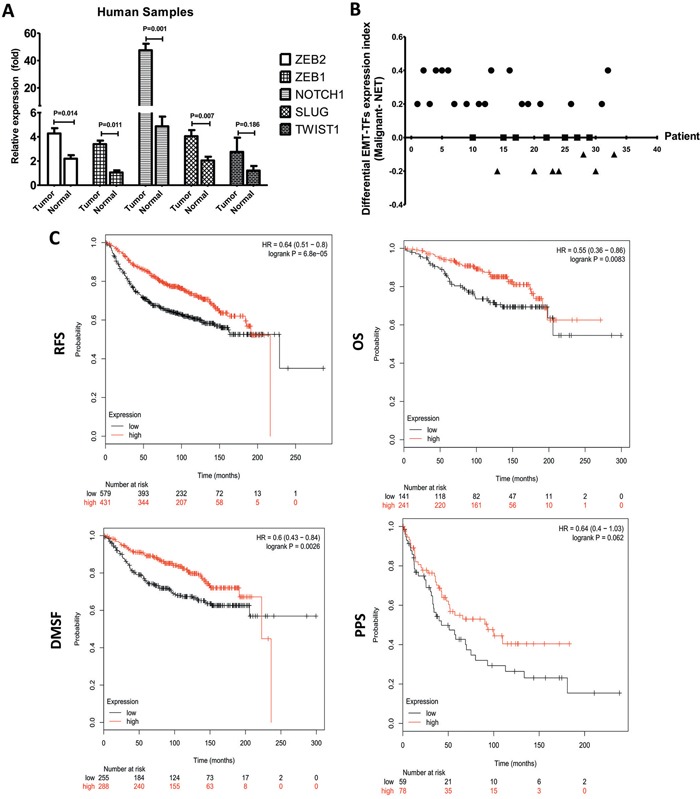
EMT-TFs are frequently upregulated in BC tissues **A**. SLUG, TWIST1 ZEB1/2 and NOTCH1 expression level were detected in MBC tissues and matched with the corresponding paired adjacent normal tissue through qRT-PCR. **B**. EMT-TFs are differentially expressed in primary malignant BC tissue compared with the corresponding paired adjacent normal tissue. For each case of the 33 patients, index of all EMT-TFs (SLUG, TWIST1, and ZEB1/2) and NOTCH1, expression (qRT-PCR score) of the primary tissue was subtracted by that of the corresponding paired adjacent normal tissue, and the resultant values (Malignant-NAT) from all 33 cases were used for plotting. The relative of EMT-TFs expression level between primary cancer and paired adjacent tissue of each case were represented as a dot. EMT-TFs are considered to be significantly upregulated, similar or negative in primary tissue when compared with the corresponding paired adjacent tissue only if the calculated index is >0 (circular dots), 0 (square dot), <0 (triangular dots), respectively. **C**. Kaplan-Meier survival analysis for the relationship between survival time and ZEB1 signature in BC was performed by using the online tool (http://kmplot.com/analysis/). All data were showed with mean ± SD. Data presented is a representative of three different experiments. The qRT-PCR results were normalized to18S. RFS, relapse free survival; OS, overall survival; DMFS, distant metastasis free survival; PPS, post progression survival.

To emphasize the role of EMT-TFs in promoting tumor metastasis, the expressions for NOTCH1, SLUG, TWIST1, and ZEB1/2 were examined by qRT-PCR from BC and their normal adjacent tissues. As the Figure 4 showed, the expressions of NOTCH1, SLUG, and ZEB1/2 were significantly increased in tissue samples compared to the matched-control group (Figure 4A). These results demonstrated a significant increase of EMT-TFs in cancer tissues. For each case of 33 patients, an index of all EMT-TFs and NOTCH1 expression of the MBC tissue (qRT-PCR score) was subtracted by that of normal tissue, and the resultant values (malignant-NAT) from all 33 cases were presented in Figure 4B. Of the 33 cases examined, 19 (57.58%) showed significantly higher expression of EMT-TFs in the cancerous tissues in comparison to the corresponding adjacent normal tissues; while another 7 (21.21%) showed similar levels of expression in both tissues; only 7 (21.21%) displayed a reverse expression pattern, in which EMT-TFs expression was lower in the cancer tissues than that of normal groups. So, EMT-TFs are differentially expressed in malignant BC tissue compared to the normal tissue.

We then analyzed the association between survival time and NOTCH1, TWIST1, SLUG, and ZEB1/2 with an online tool [34] to further extend the clinicopathologically-relevant context, with the exclusion of systemic treatment, endocrine therapy, and chemotherapy. The results revealed that higher ZEB1 expression was considerably associated with a worse overall survival (OS, *HR* = 0.55, *P* = 0.0083), worse relapse free survival (RFS, *HR* = 0.64, *P* = 0.001), post progression survival (PPS, *HR* = 0.64, *P* = 0.062), and distant metastasis free survival (DMFS, *HR* = 0.6, *P* = 0.0003) of BC patients (Figure 4C). There was no significant difference between SLUG, NOTCH1, ZEB2, and TWIST1 in BC patients (Table 2). These data indicated that ZEB1 could be used as the most important potential predictor of survival in the BC patients.

**Table 2:** Kaplan-Meier survival analysis for the relationship between survival time and NOTCH1, TWIST1, SLUG, and ZEB2 signature in breast cancer

EMT-TFs	RFS	OS	DMSF	PPS
**TWIST1**	0.16	0.19	0.043	0.3
**SLUG**	0.14	0.18	0.34	0.26
**ZEB2**	**0.05**	0.092	**0.039**	0.11
**NOTCH1**	**0.096**	0.14	0.32	0.18

Abbreviations: EMT-TFs, EMT-inducing transcription factors; ZEB1&2, Homo sapiens zinc finger E-box binding homeobox 1 and 2; NOTCH1, Notch homolog 1, translocation-associated; SLUG, Zinc finger protein; PPS, post progression survival; LSD, least significant difference; RFS, relapse free survival; OS, overall survival; DMFS, distant metastasis free survival.

**Figure 5:**
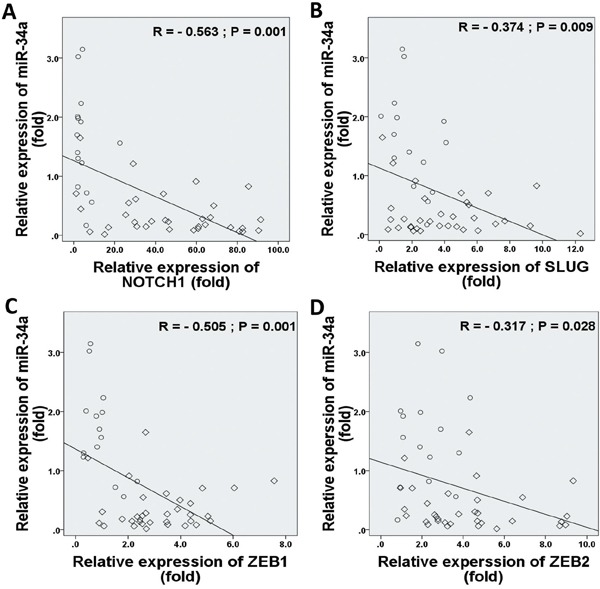
Correlations between relative expression levels of NOTCH1 A., SLUG B., ZEB1 C., and ZEB2 D. with miR-34a expression level Data indicate significantly negative associations in EMT-TFs and miR-34a expressions in MBC patients. The diamond indicates human BC specimens (n = 33), and circles represent paired adjacent normal tissue (n = 15). Data were analyzed by Spearman's rank correlation coefficient.

In considering both tumor and normal samples, miR-34a was inversely correlated with NOTCH1 (*r* = −0.563, *P* = 0.001; Figure 5A), SLUG (*r* = −0.374, *P* = 0.009; Figure 5B), ZEB1 (*r* = −0.505, *P* = 0.001; Figure 5C), and ZEB2 (*r* = −0.317, *P* = 0.028; Figure 5D) expressions. We found that TWIST1 ratios were not significantly correlated with the frequencies of miR-34a expression (*r* = −0.093, *P* = 0.532; data not shown). Therefore, we proposed that EMT-TFs over-expression might suppress miR-34a expression of BC.

### MicroRNA-34A inhibits BC progression by targeting TWIST1, NOTCH1, and ZEB1

**Figure 6:**
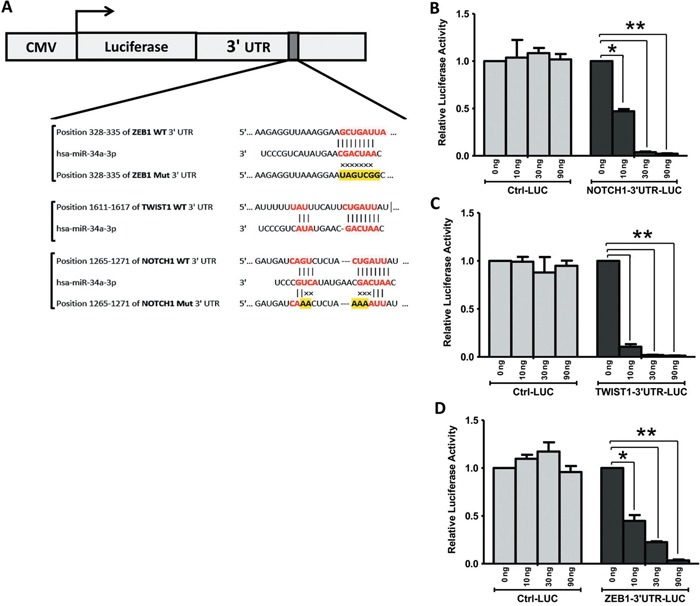
TWIST1, NOTCH1 and ZEB1 are direct targets of miR-34a **A**. miR-34a binding sequences at the 3′-UTR of NOTCH1, TWIST1 and ZEB1. Mutation was generated in the NOTCH1, and ZEB1 3′UTR sequence in the complementary site for the seed region of miR-34a. BT-549 cells were transfected with either the wild-type or mutant NOTCH1 **B**., TWIST1 **C**., ZEB1 **D**. -3′UTR luciferase reporter genes, together with different concentrations of miR-34a mimic or controls (0, 10, 30, and 90 ng). The relative luciferase values were measured and normalized by luciferase reporter assay after 48 h. NOTCH1, TWIST1, and ZEB1 expression were directly down-regulated by miR-34a. All data were showed with mean ± SD. Similar results were obtained from three independent experiments. (* *P<0.05* and ** *P<0.001* versus 0 ng treated group).

We next explored whether miR-34a is able to directly target NOTCH1, TWIST1 and ZEB1 gene expression at the post-transcriptional level by regulating the activity of mRNA (Figure 6). First, prediction on-line databases, like miR base databases, identified that NOTCH1, TWIST1 and ZEB1 were potential downstream targets of miR-34a through their interactions in the 3′-UTR region (Figure 6A). Thus, luciferase reporter assays carried out by co-transfection of different amount of pre-miR-34a (0, 10, 30, and 90 ng each well) along with NOTCH1, TWIST1 and ZEB1 wild-type 3′-UTR reporter genes respectively in BT-549 cells contributed to significant reductions in luciferase activity (Figures 6B-6D). Luciferase activities are significantly reduced in a dose dependent manner in all NOTCH1, TWIST1 and ZEB1 reporter genes. However, co-transfection of miR-34a, along with the NOTCH1, TWIST1, and ZEB1-mut 3′-UTR or control reporter genes, did not show significant effects on the luciferase activity. These results suggested that miR-34a directly targets multiple genes of NOTCH1, TWIST1, and ZEB1 *in vitro*.

### Co-delivery of miR-34A and Thymoquinone inhibits BC metastasis by regulating EMT-TFs

**Figure 7:**
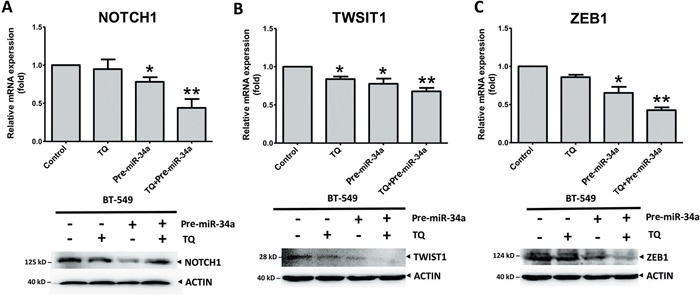
Synergic effects of thymoquinone (TQ) and miR-34a on mRNA (top panel) and protein (bottom panel) level expression of EMT-TFs TQ (5 μM) treatment for 6h for RT-PCR or 12h for Western blot pre-miRNAs (20 ng of each) treatment for 36 h inhibited the expression of NOTCH1 **A**., TWIST1 **B**., and ZEB1 **C**. in the BT-549 cell line. Results expressed as mean ± SD, and independently repeated with three times. qRT-PCR results normalized to 18S or U6 and compared with the untreated cells as a control. In western blot β-actin (ACTIN) as an internal control (* *P<0.05* and ** *P<0.001* versus control group).

Our previous investigation reported that Thymoquinone (TQ), small molecular component of *Nigella sativa*, could be used as an inhibitor of cancer growth and metastasis [35]. The epigenetic modulation of the EMT promoter is associated with TWIST1 and cadherins down-regulation and metastasis by TQ treatment in BC cells [35]. With this background, we investigated the synergistic effects of TQ and miR-34a on the expression of EMT-associated proteins. Cancer cells were treated with TQ, pre-miRNAs vector, or both. Both proteins and gene analysis showed that TQ+miR-34a significantly inhibited the mRNA level expression of NOTCH1 (*P* = 0.073; Figure 7A) TWIST1 (*P* = 0.062; Figure 7B) and ZEB1 (*P* = 0.005; Figure 7C) in the BT-549 cell line. However, no significant changes in NOTCH1 and ZEB1 expression were observed in the TQ treated groups (Figures 7A and 7C, respectively). Interestingly, the protein levels of ZEB2 and SLUG were expressed minimally or completely unexpressed in three groups in comparison to the control. In addition, some extent of reduction in ZEB2 was also observed, by TQ through either treatment or miR-34a overexpression (Supplementary Figure 3). Thus, the obtained data proposes that co-delivery of TQ and miR-34a could produce synergistic effects on tumor growth and migrations.

## DISCUSSION

The experimental investigation of this study identified a novel regulatory mechanism of EMT-TFs using miR-34a. Our results indicated that miR-34a inhibits TWIST1, ZEB1, and NOTCH1 expression upon their 3′-UTR activity in BC cells and reduces the metastatic and invasive features of MBC effectively. We also revealed that miR-34a expression is down-regulated in MBC tissues compared to normal tissues. Moreover, we found a significant inverse correlation between miR-34a expression and EMT-TFs expression. The forced miR-34a expression in MBC cell line BT-549 powerfully inhibits cell migrations and invasion. Importantly, miR-34a does not have any inhibitory effects on SLUG and ZEB2 in BC cells we investigated. We therefore presented the first comprehensive report of epigenetic inactivation of the EMT-TFs/miR-34a pathway in human BC that can potentially alter the stability of these regulations in EMT and mesenchymal state, consequently contributing to metastasis.

**Figure 8:**
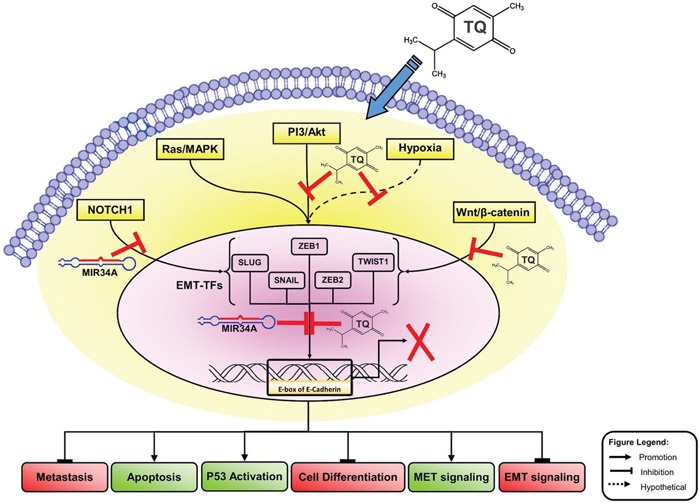
Molecular model of BC metastasis suppressed via TQ/miR-34a-initiated signaling pathways This schematic carton shows the major triggering of EMT-inducing signaling pathways, like Wnt proteins (NOTCH), Ras/MAPK kinas, PI3-kinase/Akt and hypoxia (ROS). Co-delivery of TQ/miR-34a leads to repression of metastatic, cell differentiation, and EMT factures in front of inducing of apoptosis and TP53 activations and MET features of BC cell. For more details see manuscript text. TQ, Thymoquinone; miR-34a, microRNAs-34a; PI3K/AKT, phosphatidylinositol 3-kinase (PI3K)/Akt; ROS, reactive oxygen species; MAPK, Mitogen-Activated Protein Kinase; ZEB, zinc-finger E-box-binding; EMT, epithelial to mesenchymal transitions, MET, mesenchymal to epithelial transitions; EMT-TFs, EMT-inducing transcription factors.

The underlying mechanisms governing MBC remained largely undefined, with few studies proposing the inhibitions of EMT-TFs/miR-34a feedback loop as a model for the dissemination process. Several signaling pathways, such as NOTCH1, TGF-β-SMAD, and Wnt/β-catenin, initiate the reprogramming of gene expression and transcriptional changes during EMT [10, 13, 33]. These signaling pathways, along with activating secondary pathways, like PI3K–AKT, RAS/MAPK, p38 MAPK and JUN N-terminal kinase, regulate the pathogenesis and progression of tumor as they control cell proliferation, migrations, differentiation, apoptosis, and angiogenesis [36, 37] (Figure 8). Dysregulation of these signaling pathways and aberrant activations of target genes in the EMT-inducing signaling pathways contribute to MBC progression [38, 39]. The inactivation of EMT-TFs by the human tumor suppressor micro-RNAs is a new epigenetic-targeted therapy for MBC. Consequently, several miRNAs, including miR-1 [40], miR-34a [30, 41, 42], miR-100 [43], miR-125b [44], and miR-142 [45] have been proposed to inhibit or reduce the EMT process during the advanced stage of BC [46]. A number of studies have suggested that miR-34a is potentially a regulatory microRNA that is usually up-regulated in breast, pancreatic, renal and gastric cancer [47–50]. Nevertheless, these reports on the interaction between miR-34a expression and the inactivation of EMT signaling pathway are still limited [46].

Information about regulators and mediators of EMT-TFs/miR-34a loops is still murky and unknown. Our study seeks to address that miR-34a expression is lower in human BC tissues than in normal breast tissues. Studies indicated that frequencies of miR-34a expression in the BC are controversial [51–53]. The downregulation of miR-34a expression in breast could be associated with the frequent deletion of chromosome 1p36, TP53 mutations and CpG methylation of the miR-34a promoter in these tumors [52, 54]. MiR-34a was inactivated by aberrant CpG methylation in multiple types of cancer; but breast tumors were not investigated yet [52]. A number of studies have concluded that knockdown of some EMT-TFs in cancer cell lines interacting with miR-34a inhibits cancer progression and metastasis [19, 33, 55, 56]. Now, the details of how miR-34a physically interacts with EMT-TFs and how this interaction inhibits MBC remain unclear. It is possible that miR-34a may interact with 3′-UTR of EMT-TFs by stem-loop structure and change the conformation of the binding and landing site of other EMT-activation proteins, possibly inhibiting the protein expression of EMT and disrupting the post transcriptional modification pattern through methylations and ubiquitin-proteasome pathway [57]. This hypothesis needs further investigation based on epigenetic and structural analyses. Despite this, it appears that the reduction of EMT-features and their modifications through the interaction between miR-34a and EMT-TFs may be a major functional mechanism of miR-34a in our present study.

To our knowledge, the correlation between downregulation of miR-34a expression and EMT-TFs regulations in BC was investigated systematically in this study for the first time. Despite some limitations in sample size, the current data showed an inverse correlation between miR-34a expression and EMT-TFs expression in MBC tissues, indicating that miR-34a could be a promising and novel noninvasive biomarker in BC diagnosing. There is some evidence supporting this finding, showing the negative correlations of miR-34a with the biomarkers of most cancers. Survival time and clinicpathological analysis results showed that the strongest association of ZEB1 expression is correlated with EMT and BC metastasis, raising the question on how ZEB1 and NOTCH1 induces EMT signaling activation and whether ZEB1′s increased expression in BC cells is linked to an altered or disturbed EMT process.

Previous studies indicated that miR-34a significantly suppressed proliferation, invasion, and metastasis of BC cells, which correlated to the overexpression of some EMT-TFs [19, 58, 59]. Some of these results are inconsistent with our findings, or not studied in BC. *Ahn et al*. (2012) demonstrated that ZEB1 targeted miR-34a as a direct tragedian of miR-34a in human lung cancer [60]. However, all these findings indicated that miR-34a might have a significant impact on the tumorigenesis of human cancer by targeting differential EMT signaling pathways other than those in BC. It is likely the initiation binding feedback loop of EMT-TFs /miR-34a is different depending on the types of cancer cell and physiological features. All of this insufficient information could contribute to additional inconsistencies and potential selection bias. Thus, our data need to be substantiated by an appropriate prospective and comprehensive basic study.

Interestingly, this is the first report demonstrating that the co-delivery of miR-34a and TQ is able to inactivate the downstream of the EMT signaling pathway by directly targeting TWIST1 and ZEB1 *in vivo*. Thus, co-delivery of TQ and miR-34a could produce synergistic effects on tumor growth and migrations, and a nanosystem-based co-delivery of tumor suppressive miRNAs and natural compound TQ may be a promising combination for therapeutic strategy against MBC by down-regulating TWIST1, NOTCH1 and ZEB1 in the near future. Previous studies proposed that TQ induces cell cycle arrest and triggers apoptosis of tumor cells through p38, NF-κB and ERK signaling pathways [11, 35, 61–64]. It has been reported that the anti-metastatic effects of TQ are likely to be mediated by the stimulation of miRNA profiles [35, 65–68]. Targeting miRNA and TQ may deliver a new outlook of tumor metastasis suppression by targeting EMT-TFs pathways in different cancer cells [35, 69–71]. It is not yet known whether miRNA's TQ-targeting mechanism and its specifics in controlling EMT could be associated with this activity [65, 66, 71]. In our current study, we found that EMT-TFs/TQ/miR-34a axis plays critical roles in regulating MBC contribution. Based on these results, we created a molecular model demonstrating the synergistic effect of miR-34a and TQ in MBC cell line (Figure 8). Figure 8 shows the transcriptional regulators of EMT and a representation of some points of intersection at TQ/miR-34a-inducing signaling pathways. These results clearly show that TQ/miR-34a is a potential therapeutic agent for further development in the management of BC/MBC. The aforementioned outcomes might help describe a possible aim on why EMT-TFs/TQ/miR-34a contributes to MBC progression, helping in the identification of a novel therapeutic target of MBC progression.

In conclusion, we indicate that the lower level of expression of miR-34a could be a promising and novel biomarker for MBC. The epigenetic inactivation of the EMT-TFs/miR-34a pathway can potentially push the equilibrium of these regulations toward EMT, thereby contributing to metastasis. MiR-34a alone could serve as a potential therapeutic agent for MBC and, combined with TQ, they might synergistically enhance the therapeutic effects significantly.

## MATERIALS AND METHODS

### Cell culture and transfections

Two non-metastatic BC cell lines, MCF-7 and T-47D, and three MBC cell lines, BT-549, MDA-MB-231, and MDA-MB-435, were purchased from American Type Culture Collection (ATCC, Manassas, VA, USA). All these cell lines were cultured in RPMI-1640 or DMEM supplemented with 10% fetal bovine serum (Thermo Fisher Scientific, Waltham, MA, USA) and 1% penicillin–streptomycin (Sigma-Aldrich, St Louis, MO, USA) [72]. Cell cultures were incubated at 37 °C with 5% CO_2_ in a humidified incubator. In this study, TQ was purchased from Sigma-Aldrich (St. Louis, MO, USA) and suspended in dimethyl sulfoxide (DMSO). In addition, different concentrations of TQ were used to treat cancer cell lines, while DMSO was used as control. The plasmids for miR-34a of miRNA inhibitors (HmiR-AN0440-SN-5), precursor miRNA clone in non-viral vectors (HmiR0005-MR04) and respective negative controls (CmiR-AN0001-SN, CmiR0005-MR04) (GeneCopoeia, Inc, USA) were transfected into 1×10^5^ cells per well in 24 well plates (Qiagen, Hilden, Germany).

### Plasmids

The wild type DNA fragment containing part of the wild type DNA fragment of the 3′-UTR region (−1611 to +1617 from the transcriptional initiation site) of the TWIST1 gene (NM_000474.3), miRNA 3′ UTR TWIST1 target clone in luciferase reporter vector (Cat# HmiT018328-MT01) and its miRNA Target clone control vector (Cat# CmiT000001-MT01) were purchased from GeneCopoeia Co. (GeneCopoeia Inc., MA, USA). The plasmids for miR-34a of miRNA inhibitors (HmiR-AN0440-SN-5), precursor miRNA clone in non-viral vectors (HmiR0005-MR04) and respective negative controls (CmiR-AN0001-SN, CmiR0005-MR04) were purchased from GeneCopoeia, Inc, USA. The wild type DNA fragment containing part of the 3′-UTR region (−328 to +335 from the transcriptional initiation site) of the *ZEB1* gene (NM_001174096) were amplified from human genomic DNA, and inserted into the pGL3-basic (Promega, Madison, WI, USA), an empty pGL3 vector used as a control. The mutant DNA sequences of the *ZEB1*, 3′-UTR region was synthesized and inserted into pGL3-basic vector. The pGL3-hNOTCH1-Luc reporter plasmid was kind gifts from *Prof. Benjamin Purow* (University of Virginia, Charlottesville, VA, USA) [73].

### Tissue samples and clinical data

The study was approved by the Ethics Committee of the Southwest Medical University according to the Helsinki Declaration (1983 Revision) [74, 75, 76]. In total, 48 fresh or formalin-fixed, paraffin-embedded MBC cases surgical biopsy specimens were obtained from affiliated Hospital of Southwest Medical University. All cases were diagnosed and confirmed by two oncologists and pathologist according to the World Health Organization (WHO) guidelines and the pTNM Union for International Cancer Control (UICC) pathological staging criteria. All patients signed consent and completed the questionnaire. The demographic and histopathologic variables of the all subjects such as medical, reproductive, family history, body mass index, tumor size, node status, disease stage, treatment and survival was obtained correctly according the interviewer-administered questionnaire. Based on the inclusion/exclusion criteria, the matched tumor tissues and non-tumor tissues samples comprised of 33 breast tumors, as well as normal breast tissues (n = 15) from epileptic resections. The tissues were immediately frozen in liquid nitrogen and stored at -80 °C until future RNA and protein extraction [74]. Most demographic and clinical characteristics of the subjects were compared and shown in Supplementary Table 1.

### Quantitative real-time PCR

To quantify the expression of mRNA, qRT-PCR was performed according to standard protocols as previously described [35, 77]. Testing for hsa-miR-34a (Cat# 4427975) was purchased from ABI. 18S or U6 snRNA from ABI (Cat# 4427975) was set as internal control. Primers were listed in Supplementary Table 2.

### Western blot

Total cell lysate of BC cell lines and frozen biopsies of human breast tissues were corrected by ice-cold RIPA lysis buffer (Roche, Diagnostics, Mannheim, Germany). A quantity of approximately 30 μg of whole cell lysates per lane were separated using SDS polyacrylamide gels electrophoresis (SDS-PAGE) and transferred on to Immobilon PVDF membranes (Millipore, Corporation, Billerica, MA, USA) and probed with antibodies listed in the Supplementary Table 3.

### Cell growth, migration and invasion assay

The real time analysis of cell migration, growth index and invasion were performed by using a real time cell analyzer (xCELLigence RTCA DP, Roche, Germany), as previously described in detail [35, 77].

### Luciferase reporter assay

The assays were performed according to standard protocols as previously described [35, 76]. In brief, assays were carried out by transfection of 10 ng of each reporter constructs in BT-549 cells that were with 0, 10, 30, and 90 ng miR-34a precursors respectively (Thermo Fisher Scientific) in 24-well plates. Dual-luciferase activity, expressed as relative light units (RLU), was measured using a luciferase kit (Promega, Madison, WI, USA).

### Bioinformatics

Prediction potential targets of hsa-miR-34a in the 3′-UTR of EMT-TFs was found by using the bioinformatics analysis based on the target prediction tools TargetScan (http://www.targetscan.org; release 5.1), PicTar (http://pictar.mdc-berlin.de) and miRanda (http://www.microrna.org) and miRWalk (http://www.ma.uni-heidelberg). The online Kaplan-Meier data set tool was used to analyze the association between survival time and EMT-TFs (http://www.kmplot.com/analysis/) [34].

### Statistics analysis

All statistical analyses were performed using SPSS software version 21.0 (SPSS Inc., Chicago, IL, USA) with unpaired Student's t-test or otherwise stated. All graphs were produced by GraphPad Prism 5.0 for windows software (GraphPad Software Inc., La Jolla, CA, USA).

## SUPPLEMENTARY MATERIALS FIGURES AND TABLES



## Abbreviations

MBC: metastatic breast cancer
BC: breast cancer
EMT: epithelial to mesenchymal transition
ZEB: zinc-finger E-box-binding
bHLH: basic helix–loo–helix
SLUG: zinc finger protein SNAI2
EMT-TFs: EMT-inducing transcription factors
TWIST1: Twist-related protein 1
miR-34a: MicroRNAs-34a
TGF-β: transforming growth factor beta
WHO: World Health Organization
UICC: Union for International Cancer Control
RLU: relative light unit
RFS: relapse free survival
OS: overall survival
DMFS: distant metastasis free survival
TQ: Thymoquinone
PPS: post progression survival
SD: standard deviation
UTR: untranslated region
LSD: least significant difference
qRT-PCR: quantitative reverse transcriptase-PCR
ROS: reactive oxygen species.
