# Supplementary material for: MicroRNA-34a targets epithelial to mesenchymal transition-inducing transcription factors (EMT-TFs) and inhibits breast cancer cell migration and invasion
Source: Oncotarget. 2017 Feb 9;8(13):21362–79. doi: 10.18632/oncotarget.15214 (PMC5400590; doi:10.18632/oncotarget.15214)
Supplement: Supplementary file 1 [file oncotarget-08-21362-s001.pdf]

MicroRNA-34a targets epithelial to mesenchymal transition-inducing transcription factors (EMT-TFs) and inhibits breast cancer cell migration and invasion

SUPPLEMENTARY FIGURES AND TABLES

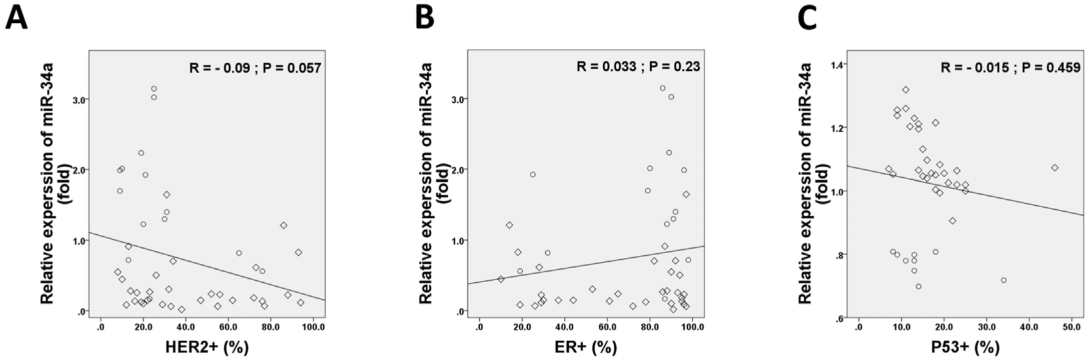

Supplementary Figure 1: Expression of HER2 A., ER B. and P53 C. Data were analyzed by Spearman's rank correlation coefficient.

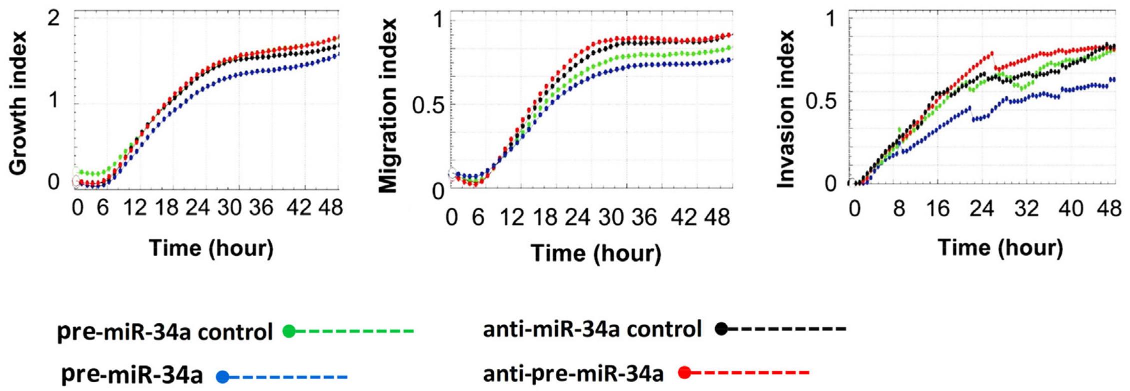

Supplementary Figure 2: Cell growth (left panel), cell migration (middle panel) and cell invasion (right panel) in MDA-MB-435 cells.

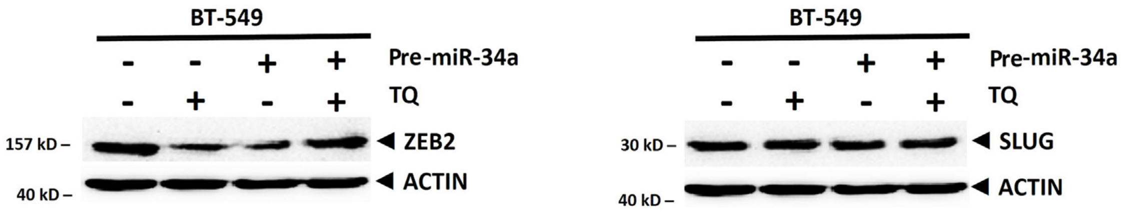

Supplementary Figure 3: Synergistic effects of thymoquinone (TQ) and miR-34a on protein level expression of ZEB2 (left panel) and SLUG (right panel) by Western blot. TQ (5 µM) treatment for 12h and pre-miRNAs (20 ng of each) treatment for 36 h in the BT-549 cell line. β-actin (ACTIN) as an internal control .

Supplementary Table 1: Demographic and baseline clinic-philological characteristics of the patients

Measure	ER +	ER -	P value
Demographic variables			
Subjects n (%)	30 (62.5)	18 (37.5)	**0.002**
Age (yrs.)	46.34 ± 1.62	47.41 ± 2.69	0.21
Histopathologic variables			
Tumor histology n (%)			
Ductal	16 (48.5%)	14 (42.4%)	0.197
Lobular	1 (3.0%)	2 (6.1%)	
Tumor Size			
≤ 3 cm	3 (17.6%)	1 (6.2%)	0.36
3-6 cm	7 (41.2%)	8 (50%)	
6-9 cm	4 (23.5%)	5 (31.2%)	
≥ 9 cm	3 (17.6%)	2 (12.5%)	
Diagnosis stage (TNM)			
Stage II	10 (30.3%)	5 (15.2%)	0.272
≥ Stage III	7 (21.2%)	11 (33.3%)	
Node status n (%)			
Negative	14 (40.0%)	12 (34.3%)	0.864
Positive	5 (14.3%)	4 (11.4%)	
Neoadjuvant therapy n (%)			
Yes	6 (18.8%)	6 (18.8%)	0.319
No	12 (37.5%)	8 (25.0%)	
Chemotherapy n (%)			
Yes	6 (18.8%)	8 (25.0%)	0.339
No	12 (37.5%)	6 (18.8%)	
PR n (%)			
Negative	4 (8.3%)	18 (37.5%)	**0.001**
Positive	26 (54.2%)	0 (0%)	
P53 n (%)			
Negative	26 (65.0%)	8(20.0%)	**0.001**
Positive	1 (2.5%)	5 (12.5%)	
Ki67 n (%)			
≤ 30	20 (41.7%)	7 (14.6%)	0.47
> 30	10 (20.8%)	11 (22.9%)	
Her2/neu n (%)			
low/weak	20 (41.7%)	7 (14.6%)	0.475
moderate/strong	10 (20.8%)	11 (22.9%)	

Abbreviations: ER, estrogens receptor; TNM, TNM classification of malignant tumours; PR, progesterone receptor; HER2, human epidermal growth factor receptor-2.
Data are presented as an number (% percent of total samples) for all others.

Supplementary Table 2: List of specific primers sequences for RT–QPCR of candidate variants

Target gene (probe No.)	Accession No.*	Sequence (5'→3')	Length	T_m	GC%
ZEB1 (#57)	NM_001174096	F, AACTGCTGGGAGGATGACAC	20	59.67	55.00
		R, TCCTGCTTCATCTGCCTGA	19	59.12	58.00
NOTCH1 (#52)	NM_017617	F, CGGGGCTAACAAAGATATGC	20	56.65	50.00
		R, CACCTTGGCGGTCTCGTA	18	59.03	61.11
SLUG (#7)	NM_003068.4	F, TGGTTGCTTCAAGGACACAT	20	57.64	45.00
		R, GCAAATGCTCTGTTGCAGTG	20	58.58	50.00
ZEB2 (#65)	NM_014795.3	F, CGATCCAGACCGCAATTAAC	20	56.98	50.00
		R, TGCTGACTGCATGACCATC	19	57.84	52.63
TWIST1 (#6)	NM_000474.3	F, GGCATCACTATGGACTTTCTCTATT	25	58.47	40.00
		R, GGCCAGTTTGATCCCAGTATT	21	57.98	47.62
18SRNA (#48)	M10098.1	F, GCAATTATTCCCCATGAACG	20	55.09	45.00
		R, GGGACTTAATCAACGCAAGC	20	57.46	50.00

Abbreviations: ZEB1&2, Homo sapiens zinc finger E-box binding homeobox 1 and 2; NOTCH1, Notch homolog 1, translocation-associated; SLUG, Zinc finger protein SNAI2; F, forward primer; R, reverse primer; Tm, optimum primer melting temperatures; GC%, guanine and cytosine percentage.

*Genbank accession number of cDNA and corresponding gene, available at http://www.ncbi.nlm.nih.gov/

Supplementary Table 3: List of specific antibodies for western blot

Epitope	Spices	Company	Catalog No.	Dilation	Source
**Primary antibodies**					
NOTCH1	Human, Mouse	eBioscience	14-5785-81	1:2000	Mouse
ZEB1	Human, Rabbit	Santa Cruz	SC-25388	1:1000	Rabbit
ZEB2	Human, Mouse	Santa Cruz	SC-271984	1:2000	Mouse
TWIST1	Human, Mouse	Abcam	ab175430	1:5000	Mouse
SLUG	Human, Mouse	Santa Cruz	SC-166476	1:3000	Mouse
ACTIN	Human, Mouse	Bioworld Technology	AA-128	1:5000	Mouse
TUBULIN	Human, Mouse	Bioworld Technology	AT819	1:5000	Mouse
**Secondary antibodies or conjugates**					
anti-mouse HRP	Mouse	Santa Cruz	G3114	1:5000	Goat
anti-rabbit HRP	Rabbit	Bioworld Technology	A0208	1:3000	Goat

Abbreviations: NOTCH1, Notch homolog 1, translocation-associated; ZEB1&2, TWIST1, Twist-related protein 1; SLUG, Zinc finger protein SNAI2; Homo sapiens zinc finger E-box binding homeobox 1 and 2; HRP, horseradish peroxidase.
